# Orthograph: a versatile tool for mapping coding nucleotide sequences to clusters of orthologous genes

**DOI:** 10.1186/s12859-017-1529-8

**Published:** 2017-02-16

**Authors:** Malte Petersen, Karen Meusemann, Alexander Donath, Daniel Dowling, Shanlin Liu, Ralph S. Peters, Lars Podsiadlowski, Alexandros Vasilikopoulos, Xin Zhou, Bernhard Misof, Oliver Niehuis

**Affiliations:** 1 Center for Molecular Biodiversity Research, Zoological Research Museum Alexander Koenig, Adenauerallee 160, Bonn, 53113 Germany; 2 Australian National Insect Collection, CSIRO National Research Collections Australia (NRCA), Clunies Ross Street, Canberra, ACT 2601 Australia; 3 Department for Evolutionary Biology & Ecology, Institute for Biology I (Zoology), University of Freiburg, Hauptstraße 1, Freiburg, 79104 Germany; 4 Institute of Molecular Biology (IMB), Ackermannweg 4, Mainz, 55128 Germany; 5 China National GeneBank, BGI-Shenzhen, Shenzhen, China; 6 Arthropod Department, Zoological Research Museum Alexander Koenig, Adenauerallee 160, Bonn, 53113 Germany; 7 Institute of Evolutionary Biology and Ecology, Zoology and Evolutionary Biology, University of Bonn, An der Immenburg 1, Bonn, 53121 Germany; 8 Beijing Advanced Innovation Center for Food Nutrition and Human Health, China Agricultural University, Beijing, 100193 China; 9 College of Food Science and Nutritional Engineering, China Agricultural University, Beijing, 100083 China

**Keywords:** Orthology, Paralogy, Splice variants, Sphecidae, Crabronidae, Transcriptome

## Abstract

**Background:**

Orthology characterizes genes of different organisms that arose from a single ancestral gene via speciation, in contrast to paralogy, which is assigned to genes that arose via gene duplication. An accurate orthology assignment is a crucial step for comparative genomic studies. Orthologous genes in two organisms can be identified by applying a so-called reciprocal search strategy, given that complete information of the organisms’ gene repertoire is available. In many investigations, however, only a fraction of the gene content of the organisms under study is examined (e.g., RNA sequencing). Here, identification of orthologous nucleotide or amino acid sequences can be achieved using a graph-based approach that maps nucleotide sequences to genes of known orthology. Existing implementations of this approach, however, suffer from algorithmic issues that may cause problems in downstream analyses.

**Results:**

We present a new software pipeline, Orthograph, that addresses and solves the above problems and implements useful features for a wide range of comparative genomic and transcriptomic analyses. Orthograph applies a best reciprocal hit search strategy using profile hidden Markov models and maps nucleotide sequences to the globally best matching cluster of orthologous genes, thus enabling researchers to conveniently and reliably delineate orthologs and paralogs from transcriptomic and genomic sequence data. We demonstrate the performance of our approach on *de novo*-sequenced and assembled transcript libraries of 24 species of apoid wasps (Hymenoptera: Aculeata) as well as on published genomic datasets.

**Conclusion:**

With Orthograph, we implemented a best reciprocal hit approach to reference-based orthology prediction for coding nucleotide sequences such as RNAseq data. Orthograph is flexible, easy to use, open source and freely available at https://mptrsen.github.io/Orthograph. Additionally, we release 24 *de novo*-sequenced and assembled transcript libraries of apoid wasp species.

**Electronic supplementary material:**

The online version of this article (doi:10.1186/s12859-017-1529-8) contains supplementary material, which is available to authorized users.

## Background

Inferring the evolution of gene families, the phylogeny of species, and tracing the biogeography of populations depend on reliable delineation of orthologous genes and paralogous copies of them. While delineation and identification of orthologous and paralogous genes has been firmly established for studying genomic data (reviewed by [1] and benchmarked by [2]), few approaches are currently available for assessing transcripts in the same manner (proposed by, e.g., [3] and [4]). Each of these approaches exhibits, and suffers from, specific problems, potentially leading to erroneous species and gene tree inference (see below). We developed a novel software pipeline, called Orthograph, for convenient, fast, and reliable identification of orthologous (and paralogous) nucleotide or amino acid sequences, which resolves existing algorithmic and software-technical issues. Orthograph builds on previously proposed graph-based clustering algorithms, but extends them without sacrificing accuracy or computational speed.

When comparing the gene repertoires of species, one of the first analytical steps is the delineation of orthologous genes (*orthologs*), i.e., the identification of genes that originated from a single gene in the last common ancestor of the compared species. Each of the delineated orthologous groups (OGs) can also include species- or lineage-specific gene copies (*inparalogs*), that evolved by gene duplication after the evolutionary split of the ancestor into different species [5]. Finally, horizontal gene transfer can give rise to xenologous gene copies (*xenologs*) from a single ancestral gene [5].

Two fundamentally different approaches to identify potential orthologs, paralogs, and xenologs have been established: tree-based and graph-based approaches. The benefit of graph-based approaches, which we will subsequently focus on, is their computational efficiency and scalability (for reviews and a comprehensive discussion of the benefits of the different approaches, see [6] or [1]). In general, graph-based approaches assessing gene orthology make use of the genome-wide best reciprocal hit (BRH) criterion. It rests on the assumption that orthologs in two genomes are more similar to each other than to any other gene in the compared genomes, since they are direct and exclusive descendants from a single ancestral gene [7].

Various graph-based approaches based on the BRH criterion have been developed that *de novo* infer orthology among genes and proteins in the gene or protein sets of sequenced and annotated organisms, such as OrthoMCL [8], COCO-CL [9], OrthoDB [10], InParanoid [11], OrthoFinder [12], and OMA [13]. The reliability of these methods critically depend on the fact that differential gene loss is the exception and that gene or protein repertoires are complete. This means that in order to apply a graph-based approach to infer gene orthology among genomes, the organisms’ gene or protein repertoire must be reliably known. These methods are therefore not appropriate for assessing orthology among nucleotide sequences in sequenced transcriptomes, since transcript libraries contain only a subset of the organisms’ actual gene repertoire. The nucleotide sequence of a gene may be missing in a given transcript library simply because the gene was not (sufficiently highly) expressed at the time of RNA preservation. Given that transcriptome sequencing represents an extremely valuable and cost-efficient strategy to sample coding nucleotide sequences of a large fraction of an organism’s gene repertoire [14], several graph-based approaches have been developed that are dedicated to ortholog identification in transcript libraries.

A possible solution to the aforementioned problem in transcript orthology assessment is to assign transcripts to OGs whose genealogical relationships have already been reliably inferred, rather than to infer orthology of these genes *de novo* from the transcripts. Knowledge of the genealogical relationships of genes can be derived from comparative genomic analyses and may be retrievable from public databases such as OrthoDB [10]. This approach has been implemented in OrthoSelect [4] and HaMStR [3]. However, OrthoSelect does not implement the BRH criterion, but a unidirectional search. OrthoSelect is thus prone to false positives. HaMStR, on the other hand is more sophisticated since it applies a BRH orthology prediction strategy. Specifically, HaMStR uses profile hidden Markov models (pHMMs) that represent properties of the aligned amino acid sequences of each known OG to search a transcript library on the amino acid level for matches. All retrieved hits are then searched against the entire set of proteins, i.e., the proteome (also referred to as “official gene set”) as reference gene set (RGS), of each of the species of which amino acid sequences were used to construct the pHMM. If this reciprocal search retrieves the same amino acid sequence(s) that was (were) used in the construction of the pHMM, a the respective transcript is mapped to the OG in question.

The algorithm of HaMStR is “memoryless”, meaning that during evaluation of the BRH criterion for a given OG, it does not consider which transcripts have been assigned to other OGs. Since transcripts are assigned to OGs on a per-OG basis without considering results from evaluations for other OGs and keeping track of what transcripts have already been assigned, it is possible that a given transcript is mapped to more than one gene. This issue of redundant transcript assignments can result in a misled inference of phylogenetic relationships, as has been shown [15, 16], and can potentially compromise downstream analyses. In HaMStR, it would be conceivable to prevent redundant transcript assignment by implementing a record of previously assigned transcripts. However, such a first-come-first-serve approach cannot be justified: transcripts must be assigned to the OG that they are most likely orthologous to, not to the OG that came first in the search order. Since this serious issue cannot be solved using the HaMStR algorithm, we developed Orthograph: a different algorithm that circumvents redundant transcript assignments and instead maps transcripts to the globally best matching OG.

To assess the sensitivity and accuracy of Orthograph, we tested whether or not Orthograph a) reliably identifies orthologs, b) detects known paralogs, and c) finds known isoforms or alternative transcripts. We additionally searched 24 *de novo*-sequenced transcript libraries of apoid wasps for 5561 orthologous genes to assess the computational performance of Orthograph. Finally, we verified that Orthograph does not map transcripts to more than one gene by re-analyzing a dataset that has been processed with HaMStR. Our results demonstrate that Orthograph’s performance is on par with HaMStR’s while not suffering from redundant transcript assignment. Further, we emphasize the flexibility of Orthograph and highlight features that are likely of particular interest for a wide array of analyses in molecular evolutionary biology and in comparative genomics in particular.

## Implementation

The Orthograph software package is divided into three main tools that handle (i) database management (manager), (ii) forward and reverse searches (analyzer), and (iii) clustering of orthologous transcripts and output (reporter). The separation into three distinct tools is a deliberate design choice to address work environments where users do not have full administrative privileges. This facilitates implementation in a high-performance computing cluster setup where the administrator can use the appropriate tool to manage the database, while users only need to run the actual analysis tools. In addition, this design allows the user to evaluate the alignment search results using different settings (e.g., different alignment bit score thresholds to fine-tune and optimize parameters) quickly without re-running the computationally expensive searches.

**Fig. 1:**
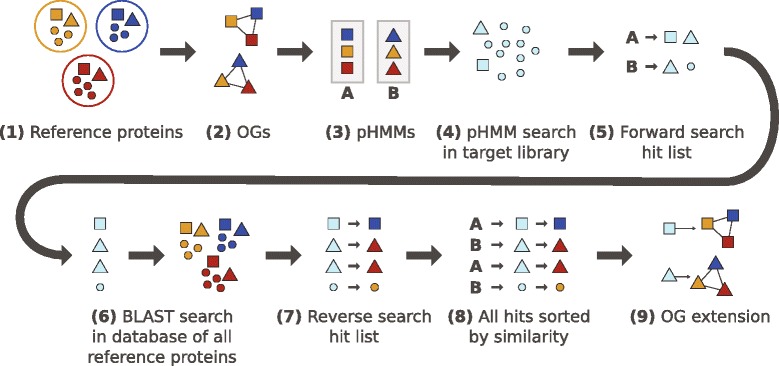
Orthograph workflow. From a set of reference proteins (*1*), the proteins are clustered to form orthologous groups (OGs) (*2*). These OGs are aligned to construct profile hidden Markov models (pHMMs) (*3*). The pHMMs are used to search for candidate orthologs in the target library (*4*). Each of the obtained hit amino acid sequences (*5*) is used as a query for a BLAST search in a database comprising all reference proteins (including the ones forming OGs) (*6*). Search results from both forward and reverse searches (*7*) are collated and sorted by bit score, with the reverse search result order being subordinated to the forward result order (*8*). This list is evaluated in descending order: if the reverse search hit a protein that is part of the OG used for the forward search, the candidate ortholog is mapped to the OG (*9*)

Orthograph builds on the transcript orthology assessment strategy via BRH suggested by [3]. In contrast to the implementation of this strategy in HaMStR, Orthograph assigns a given transcript to the *globally* best matching OGs while making sure that no transcript is assigned more than once. It additionally identifies all transcripts (splice variants and inparalogs) present in an assembled transcript library that are putatively homologous to a given OG. The specific transcript orthology assignment algorithm is as follows (Fig. 1); note that steps 1 through 3 are only required once since their output can be used for all subsequent analyses:

1. The proteomes (“reference gene sets”, RGS) of reference species are used as input.
2. Orthologous genes from all reference proteomes are clustered to form orthologous groups (OGs). This information is provided from public databases or one’s own orthology delineation in the RGS.
3. For each OG, the amino acid sequences are aligned and the multiple sequence alignment (MSA) is used to construct a profile HMM.
4. These pHMMs are used to search the transcript sequences on the amino acid level for candidate homologs.
5. Search results are stored in a relational database.
6. For each pHMM search hit, the target amino acid sequence section matching the pHMM is used as a query to search in a database that includes all genes from the RGS (including the genes that form OGs) on the amino acid level.
7. The results of the reverse search are also stored in the relational database.
8. After all forward and reverse searches have completed, the clustering of BRH pairs takes place: search results from all forward searches are sorted by descending alignment bit score. For each forward alignment search result, the corresponding reverse alignment search results are sorted by descending alignment bit score as well. They are evaluated in order of descending alignment bit score for the forward search results, starting with the highest alignment bit score.
9. If the best reverse search hit of a given transcript is part of the OG that the pHMM for the forward search is based on (i.e., the BRH criterion is fulfilled), the target transcript is assigned to the OG. The target transcript section is marked so that it cannot be assigned again. Each entry in the database is evaluated in this manner.

Orthograph performs several post-processing steps on transcripts assigned to OGs. By aligning the transcript fulfilling the BRH criterion to the most similar orthologous amino acid sequence of a reference species using Exonerate [17], it infers a frameshift-corrected open reading frame (ORF). Orthograph allows to extend the ORF beyond the pHMM alignment sequence section for which the BRH criterion was fulfilled while making sure that the orthologous region is covered by a user-defined percentage of the ORF length. Subsequently, it provides both the amino acid sequence and the exactly corresponding frameshift-corrected nucleotide sequence of a given transcript. Additionally, Orthograph can concatenate transcripts of a given OG to simplify downstream analyses (e.g., phylogenomic investigations). In all above analysis steps, the user can fine-tune all relevant search and evaluation parameters using configuration files for clarity, documentation, and reproducibility.

Orthograph has been developed with user friendliness in mind. As a result, it is easy to install and runs on any Unix/Linux system (including OS X) that provides its dependencies (see Materials and Methods). The generation of custom-tailored ortholog sets, e.g., from public databases is facilitated by its ability to parse simple tab-delimited tables. Input from public databases such as OrthoDB is easily formatted accordingly using standard UNIX or spreadsheet tools. In addition, the Orthograph package contains helper scripts that simplify the preparation of RGS sequence files for custom-made ortholog sets as well as summarize results for multiple analyses, e.g., different species or using different settings.

When designing a custom ortholog set, users should pay close attention to the taxon sampling. Genes that occur in at least two species in each OG are recommended so that the resulting pHMMs are more informative than when based on single sequences only. In terms of OG number, there is no lower or upper bound since the selection depends on the research question. Orthograph runtime increases linearly with each additional OG.

Detailed methods, data sources as well as system requirements are listed in the Additional file 1 (Figures S1–S5, Tables S1–S3).

## Results and discussion

### Sensitivity and accuracy when searching for single-copy orthologs

To assess the sensitivity and accuracy of Orthograph, we employed it to identify genes of known orthology in the RGS of the honeybee, *Apis mellifera* (15,314 genes, [18]), and Jerdon’s jumping ant, *Harpegnathos saltator* (18,564 genes, [19]). Specifically, we searched the RGS for 4625 protein-coding genes provided by OrthoDB 5 [20] as being single-copy across four species of Hymenoptera (*Apis mellifera* [18], *Camponotus floridanus* [19], *Harpegnathos saltator* [19], *Nasonia vitripennis* [21]) and the outgroup beetle *Tribolium castaneum* [22] (download URLs are listed in the Additional file 1: Table S3). Note that we removed all entries of the respective taxon whose RGS we analyzed for assessing the sensitivity and accuracy of Orthograph from this ortholog set (resulting in two sets: one without entries from *A. mellifera*, and one without entries from *H. saltator*). Of the 4625 protein-coding genes that we searched for, Orthograph identified 4582 (99.07%) in the RGS of *A. mellifera* and 4590 (99.24%) in the RGS of *H. saltator* (Table 1). In the case of *A. mellifera*, five proteins were assigned to other OGs than they were assigned by OrthoDB. We found a similar result for three proteins of the RGS of *H. saltator*. Visual inspection of these proteins suggested that the orthology assignment of these proteins in the OrthoDB database is not correct (for an in-depth assessment and discussion of an example see Additional file 1: Figure S5). The low fraction (less than 1%) of non-recalled genes were caused by a comparable effect (Figure S5). Thus, the sensitivity (true positive rate), defined as the ratio of true positives to true positives plus false negatives, was 0.9896 for the *A. mellifera* RGS and 0.9918 for the *H. saltator* RGS. The accuracy, defined as the ratio of true positives plus true negatives to the total number of genes in the RGS, was 0.9965 for the *A. mellifera* RGS and 0.9978 for the *H. saltator* RGS.

**Table 1:** Results from the tests that compare Orthograph performance to HaMStR [3]

Software	Test	Genes	Species	OGS	Found	TP	FP	FN	Sens.	Acc.
Orthograph	Single-copy	4625	*A. mellifera*	15,314	4582	4577	5	48	0.990	0.996
Orthograph	Single-copy	4625	*H. saltator*	18,564	4590	4587	3	38	0.992	0.997
HaMStR	Single-copy	4625	*A. mellifera*	15,314	4589	4588	3	39	0.992	0.997
HaMStR	Single-copy	4625	*H. saltator*	18,564	4573	4571	2	54	0.988	0.996
Orthograph	Isoforms	8	*C. floridanus*	17,064	7	7	0	1	0.875	0.999
HaMStR	Isoforms	8	*C. floridanus*	17,064	7	7	0	1	0.875	0.999
Orthograph	Inparalogs	647	*A. cephalotes*	18,093	583	583	0	6	0.901	0.996

Sensitivity is defined as the ratio of true positives (TP) to TP plus false negatives (FN). Accuracy is defined as the ratio of TP plus true negatives (TN) to the total number of genes in the official gene set (OGS). FP, false positives. Note that the results are meant to demonstrate equality in performance despite algorithmic differences

For comparison, HaMStR v13.2.3 was run on the same datasets with comparable parameters. HaMStR identified 4589 genes (99.22%) in the RGS of *A. mellifera* (1 false positive) and 4573 genes (98.88%) in the RGS of *H. saltator* (2 false positives). This results in a sensitivity of 0.992 in the *A. mellifera* RGS and of 0.9883 in the *H. saltator* RGS, and an accuracy of 0.9975 in the *A. mellifera* RGS and of 0.9969 in the *H. saltator* RGS.

The input data on ortholog relations were retrieved from OrthoDB which contains OG information inferred in a purely automated fashion [20]. OrthoDB has been attested low numbers of false positives and spurious assignments [2]; the proportion of less than 1% of the genes that were recalled wrongly by Orthograph are in line with these benchmarks. Orthograph and HaMStR perform roughly equally in accuracy and sensitivity when it comes to identifying single-copy orthologs.

### Identification of splice variants or isoforms

We used Orthograph to assess orthologous amino acid sequences including isoforms in the RGS of the Florida carpenter ant, *Camponotus floridanus*, a species whose genes and corresponding proteins are part of the ortholog set analyzed before (see above). In the *C. floridanus* RGS, eight genes that are part of the ortholog set each encode an alternative isoform. Orthograph readily assigned the alternative isoforms of seven of these genes to the correct OGs. In the remaining gene, however, the amino acid sequence of the isoform that Orthograph could not find was very short (46 amino acids) in length. Only 21 of the 46 amino acid sites can be well aligned to the OG and were identified as BRH. It is possible that amino acid sequences that are significantly shorter than the majority of the OG are scored poorly by the pHMM search and/or the subsequent reverse search so that they eventually do not fulfill the BRH criterion and are not recognized by Orthograph.

HaMStR, in comparison, also identified all isoforms of seven of the eight genes correctly. However, it reports them as co-orthologs. Strictly speaking, this term is only correct when, while searching for single-copy orthologs, one or more copies of the same gene are identified. Orthograph, in addition to reporting, provides tabular output with alignment coordinates, HMM alignment bit scores and e-values for further statistical analyses.

While it would be highly desirable for users to also obtain information on the occurrence of different isoforms (or alternative transcripts on the transcriptional level) in different species, alternative transcripts are difficult to distinguish from transcripts of inparalogs or from transcript assembly artifacts without additional information, for example on the genealogy of the species, whose transcript libraries have been investigated, and/or on the transcript’s expression level. However, Orthograph provides tabular output files that can facilitate corresponding downstream analyses. Specifically, the Orthograph output files inform about a) what transcripts form BRHs with ortholog groups and b) what transcripts assigned by Orthograph to the same ortholog group overlap (i.e., partially refer to the same coding sequence) and could thus represent alternative transcripts (or assembly artifacts).

#### Protein isoforms and splice variants in the reference ortholog set can lead to systematic errors and false positives

The presence of isoforms and splice variants in an RGS dataset can lead to wrong clustering to OGs and/or false negatives (discarded sequences that should have been mapped elsewhere). Because it is impossible to know in advance which isoform of a gene or transcribed gene is present in a given transcript library, it is likely that a BRH search will fail if more than one highly similar amino acid sequence are present in the reference RGSs. This occurs because the best reverse search hit of a candidate ortholog against the database comprising all proteins in an RGS may return an isoform of the protein that was not used in the pHMM, leading to a failure to fulfill the BRH criterion. Therefore, isoforms should either be removed from RGS databases prior to using them in Orthograph (or in any reference-based orthology prediction tool, for that matter), or the OGs should be extended to also include the isoforms.

### Identification of inparalogs

In order to demonstrate Orthograph’s capabilities to detect inparalogous gene copies, we used it to assess genes that are known to have inparalogous copies in the RGS of the leafcutter ant, *Atta cephalotes* [23]. Specifically, we retrieved an ortholog set from OrthoDB 5 comprising 301 OGs that contain genes that are known to be single copy in the genomes of *A. mellifera*, *C. floridanus*, *H. saltator*, *N. vitripennis*, and *T. castaneum*, but are multi-copy genes in *A. cephalotes*. These 301 OGs include altogether 647 single-copy and multi-copy genes from *A. cephalotes*: 273 are duplicated, 18 are triplicated, seven have four copies, two have six copies and one has seven copies. Orthograph readily assigned 583 of the 647 multi-copy genes to the correct OG (90.1%). Two of the 301 OGs were not assigned, one of which contained four, the other contained two gene copies. In both cases, the genes from *A. cephalotes* were much shorter than the remaining genes in the OG (18% resp. 19% of the average amino acid sequence length), possibly leading to the respective transcripts failing to fulfil the BRH criterion in the reverse search step due to an insufficient alignment length. These edge cases again highlight the importance of high-quality genome sequencing and annotation efforts, as they provide the basis for many downstream analyses, including full-length gene sequences for reference-based orthology assessment.

### Non-redundant mapping of transcripts

In order to test whether Orthograph indeed does not assign transcripts to more than one OG, we re-analyzed the dataset published by [24], who used HaMStR version 8 [3]. Orthograph assigned transcripts to 1,253 OGs, the same number as obtained by [24]. However, Orthograph found transcripts of the analyzed genes in, on average, slightly more taxa (Orthograph: 28.079, [24]: 26.699). None of the transcripts was assigned to more than one OG. In the dataset published by [24], 274 transcripts were assigned redundantly, however the orthologous regions were not overlapping. As [24] removed a total of 1.3% of their sequences from the dataset due to redundantly assigned transcripts, Orthograph yielded 1.4% more taxa per gene, leading to a denser data matrix for downstream (phylogenetic) analyses.

### Computational performance of Orthograph

To demonstrate the computational performance of Orthograph, we searched 24 apoid wasp transcriptome assemblies for 5561 selected OGs (sequence data are deposited at NCBI GenBank; accession numbers are listed in Additional file 2). The analysis time when using a single thread increases linearly with total transcriptome assembly length (Spearman rank correlation, *S*=326, *p*≪0.001, Additional file 1: Figure S3). Single-threaded analysis time also increases with the number of assembled transcripts, showing a linear trend, but no significant correlation (Spearman rank correlation, *S*=1430,*p*=0.069).

Given that next-generation RNAseq datasets tend to be large and current comparative genomic investigations analyze hundreds, if not thousands of genes (e.g., [14, 25], the 1000 plants initiative (https://sites.google.com/a/ualberta.ca/onekp/)), with a linear runtime increase Orthograph does not pose a time bottleneck for current and future large-scale studies such as the numerous group-specific subprojects of the 1KITE consortium (http://1kite.org/subprojects.html). For employment in high-performance cluster computing environments, Orthograph supports multi-threading: it offers a linear speedup of about 1x until up to four threads (Fig. S4). Orthograph scales well with a speedup of 15 to 80% per additional thread up to 12 threads. Using 16 threads reduces Orthograph running time to around 11% compared to a single-threaded analysis.

Because most of the data are stored in a relational database on the hard drive, Orthograph requires only little memory and allows to re-evaluate stored search results with different parameters, which takes only a fraction of the original analysis time. In a centralized server-client setup using the MySQL database backend, the database management overhead is solely handled by the server, freeing CPU resources for the alignment searches on the clients. For installation in a grid computing environment where adding a dedicated database server is not feasible, the SQLite database backend [26] is provided. The file-based SQLite database system can be applied anywhere thanks to its portable and performant implementation (and is installed by default in most Linux distributions and Mac OS X), thus it is the default database backend in Orthograph.

### Advantages of graph-based orthology prediction strategies

Orthograph uses a graph-based approach, like HaMStR and OrthoSelect as well as orthology prediction tools that assess orthology among genes in completely sequenced and annotated genomes, such as OrthoMCL, OrthoDB, OMA, or InParanoid. In contrast, tree-based orthology prediction strategies such as TreeFam, Ensembl Compara, or the one implemented in [27], employ an algorithm that reconciles a phylogenetic tree topology of a gene or gene set with the topology of the respective species phylogenetic tree. This requires a) a multiple sequence alignment (MSA) of a gene’s amino acid or nucleotide sequences, and b) a phylogenetic tree inference. Both steps are not only computationally expensive, but also introduce additional sources of bias at each step. The much reduced computational complexity of a bidirectional alignment search compared to a phylogenetic tree inference enables Orthograph to run on standard workstation computers without necessitating a high-performance computing environment. A number of graph-based and tree-based orthology assessment methods have been reviewed by [2].

### Reference-based orthology search accuracy depends on reference database quality

Reference-based algorithms for assessing transcript orthology can only be as accurate as the content of the database providing reference OGs. The results from testing the performance of Orthograph affirm that reference-based orthology prediction requires adequate orthology delineation in reference genomes. These findings further highlight the necessity for reliable identification of ortholog relations in completely sequenced genomes as well as continuously updated databases such as OrthoDB that lay the foundation for a plethora of downstream comparative analyses. In order to provide comprehensive information, these databases require high-quality genomic data as well as reliable structural and functional gene annotation; thus, the importance of continued genome sequencing and rigorous annotation efforts must not be underestimated. Likewise, many assembled (draft) genomes are far from complete in terms of having properly identified their *actual* gene content [28], which also hinders reliable inference of orthology among them.

### Reciprocal search by using HMMER and BLAST

Orthograph makes use of both pHMM-based and BLAST search technology. By combining these two fundamentally different alignment search algorithms, it draws considerable sensitivity and accuracy. Profile HMM-based similarity searches have been shown to be more sensitive than BLAST when it comes to detecting remotely related sequences [29]. By restricting the reverse BLAST search to only the (sub)sequence that was found to be putatively homologous during the pHMM search, the BLAST query becomes more informative. Therefore, the practice of using BLAST for the reverse search in Orthograph improves confidence in the subsequent orthology hypothesis by applying a conservative search criterion. For an illustration of the interrelations between the search results and their respective subsequences, see Additional file 1: Figures S1 and S2.

BLAST uses a heuristic algorithm and does not guarantee an optimal local alignment. To also support a non-heuristic Smith-Waterman algorithm, we have, in addition to BLAST, implemented SWIPE [30], which is also used in OrthoDB. SWIPE uses a BLAST database, thus the BLAST package is required to generate the database; however the SWIPE search algorithm does not result in inconsistencies that are possible with BLAST’s alignment heuristic. Users can opt to use the SWIPE algorithm with appropriate configuration settings.

### Limits of the methods

Orthograph is intended to map transcripts of a single species to reference OGs. Orthology or paralogy relations between genes of more than one species cannot be established using transcriptomic datasets as they are inherently incomplete. For assessing orthology among genes in completely sequenced and annotated genomes, specialized tools exist, such as OrthoMCL [8], InParanoid [11], or the OrthoDB toolset, which is now public [10]. Additionally, alternative transcripts or splice variants are difficult to distinguish in a *de novo* transcriptome assembly without additional read coverage data, which is why Orthograph refrains from explicitly predicting them. Orthograph does, however, report transcripts that are potential alternative transcripts or splice variants in order to allow researchers to further investigate them.

## Conclusion

With Orthograph, we provide a software solution to accurately assign transcripts (and other coding sequences) to known groups (clusters) of orthologous genes (OGs). Orthograph maps transcripts to the globally best matching OG, circumventing the problem of redundantly assigning transcripts to more than one OG. With its specific algorithm, Orthograph solves this issue that earlier implementations of graph-based BRH mapping strategies suffered from, while maintaining the high sensitivity and accuracy of the BRH approach. We developed Orthograph to be an asset in many fields by offering additional functionality compared to earlier implementations of graph-based BRH mapping strategies. Orthograph is easy to install and use and thereby facilitates comparative analyses of transcriptomic and other coding sequence data. It was furthermore designed to point users to possibly existing alternative transcripts and paralogous genes, thereby significantly broadening the scope of the software. The wide applicability of Orthograph has been demonstrated by its application in a phylogenomic study on apoid wasps using target DNA sequencing baits [31] and the numerous subprojects of the international 1KITE project, which investigate intraordinal phylogenetic relationships of insects. Orthograph provides researchers with a convenient, performant, general-purpose tool for analyses in a plethora of disciplines in evolutionary biology.

## Availability and requirements

**Project name:** Orthograph;**Project home page:** https://mptrsen.github.io/Orthograph;**Operating system(s):** Linux/OS X;**Programming language:** Perl, SQL;**Other requirements:** See Additional file 1: Table S1.

## Additional files

Additional file 1 Supplemental methods and data tables. **Figure S1**. Alignment regions in Orthograph; **Figure S2**. ORF extension criteria; **Figure S3**. Orthograph runtime is significantly correlated to total transcriptome assembly length; **Figure S4**. Speedup plot for multi-threaded analysis; **Figure S5**. Example multiple sequence alignment of an OG to demonstrate a possible assignment of a transcript to the “wrong” OG. (PDF 1020 kb)

Additional file 2 Species, 1KITE library IDs (see http://1kite.org/1kite_species.php), number of assembled transcripts, total assembly size, N50 values, and NCBI GenBank accession numbers. Note that the assemblies were filtered to contain only contigs longer than 199 bp. (TXT 4 kb)

## Abbreviations

BRH: Best reciprocal hit
MSA: Multiple sequence alignment
OG: Ortholog group
ORF: Open reading frame
pHMM: Profile hidden Markov model
RGS: Reference gene set

## References

[1] Kristensen DM, Wolf YI, Mushegian AR, Koonin EV. Computational methods for gene orthology inference. Brief Bioinformatics. 2011; 12(5):379–91. doi:10.1093/bib/bbr030. Accessed 11 Oct 2012. 10.1093/bib/bbr030 PMC3178053 21690100

[2] Trachana K, Larsson TA, Powell S, Chen WH, Doerks T, Muller J, Bork P. Orthology prediction methods: A quality assessment using curated protein families. BioEssays. 2011; 33(10):769–80. doi:10.1002/bies.201100062. Accessed 24 Jan 2012. 10.1002/bies.201100062 PMC3193375 21853451

[3] Ebersberger I, Strauss S, Von Haeseler A. HaMStR: Profile hidden markov model based search for orthologs in ESTs. BMC Evol Biol. 2009; 9(1):157. doi:10.1186/1471-2148-9-157. 10.1186/1471-2148-9-157 PMC2723089 19586527

[4] Schreiber F, Pick K, Erpenbeck D, Wörheide G, Morgenstern B. OrthoSelect: A protocol for selecting orthologous groups in phylogenomics. BMC Bioinformatics. 2009; 10(1):219. doi:10.1186/1471-2105-10-219. Accessed 15 Nov 2014. 10.1186/1471-2105-10-219 PMC2719630 19607672

[5] Koonin EV. Orthologs, paralogs, and evolutionary genomics. Annu Rev Genet. 2005; 39:309–38. doi:10.1146/annurev.genet.39.073003.114725. 10.1146/annurev.genet.39.073003.114725 16285863

[6] Dutilh BE, van Noort V, van der Heijden RTJM, Boekhout T, Snel B, Huynen MA. Assessment of phylogenomic and orthology approaches for phylogenetic inference. Bioinformatics. 2007; 23(7):815–24. doi:10.1093/bioinformatics/btm015. Accessed 24 Aug 2011. 10.1093/bioinformatics/btm015 17237036

[7] Altenhoff AM, Studer RA, Robinson-Rechavi M, Dessimoz C. Resolving the ortholog conjecture: Orthologs tend to be weakly, but significantly, more similar in function than paralogs. PLoS Comput Biol. 2012; 8(5):1002514. doi:10.1371/journal.pcbi.1002514. Accessed 16 Jan 2013. 10.1371/journal.pcbi.1002514 PMC3355068 22615551

[8] Li L, Stoeckert CJ, Roos DS. OrthoMCL: Identification of ortholog groups for eukaryotic genomes. Genome Res. 2003; 13(9):2178–89. doi:10.1101/gr.1224503. 10.1101/gr.1224503 PMC403725 12952885

[9] Jothi R, Zotenko E, Tasneem A, Przytycka TM. COCO-CL: Hierarchical clustering of homology relations based on evolutionary correlations. Bioinformatics. 2006; 22(7):779–88. doi:10.1093/bioinformatics/btl009. Accessed 18 Jan 2013. 10.1093/bioinformatics/btl009 PMC1620014 16434444

[10] Kriventseva EV, Tegenfeldt F, Petty TJ, Waterhouse RM, Simao FA, Pozdnyakov IA, Ioannidis P, Zdobnov EM. OrthoDB v8: Update of the hierarchical catalog of orthologs and the underlying free software. Nucleic Acids Res. 2015; 43(D1):250–6. doi:10.1093/nar/gku1220. Accessed 26 Feb 2015. 10.1093/nar/gku1220 PMC4383991 25428351

[11] Sonnhammer ELL, Östlund G. InParanoid 8: Orthology analysis between 273 proteomes, mostly eukaryotic. Nucleic Acids Res. 2015; 43(Database issue):234–9. doi:10.1093/nar/gku1203. 10.1093/nar/gku1203 PMC4383983 25429972

[12] Emms DM, Kelly S. OrthoFinder: Solving fundamental biases in whole genome comparisons dramatically improves orthogroup inference accuracy. Genome Biol. 2015; 16(1):157. doi:http://dx.doi.org/10.1186/s13059-015-0721-2. Accessed 8 Jan 2016. 10.1186/s13059-015-0721-2 PMC4531804 26243257

[13] Altenhoff AM, Škunca N, Glover N, Train CM, Sueki A, Piližota I, Gori K, Tomiczek B, Müller S, Redestig H, Gonnet GH, Dessimoz C. The OMA orthology database in 2015: Function predictions, better plant support, synteny view and other improvements. Nucleic Acids Res. 2015; 43(D1):240–9. doi:10.1093/nar/gku1158. Accessed 3 Sept 2015. 10.1093/nar/gku1158 PMC4383958 25399418

[14] Misof B, Liu S, Meusemann K, Peters RS, Donath A, Mayer C, Frandsen PB, Ware J, Flouri T, Beutel RG, Niehuis O, Petersen M, Izquierdo-Carrasco F, Wappler T, Rust J, Aberer AJ, Aspock U, Aspock H, Bartel D, Blanke A, Berger S, Bohm A, Buckley TR, Calcott B, Chen J, Friedrich F, Fukui M, Fujita M, Greve C, Grobe P, Gu S, Huang Y, Jermiin LS, Kawahara AY, Krogmann L, Kubiak M, Lanfear R, Letsch H, Li Y, Li Z, Li J, Lu H, Machida R, Mashimo Y, Kapli P, McKenna DD, Meng G, Nakagaki Y, Navarrete-Heredia JL, Ott M, Ou Y, Pass G, Podsiadlowski L, Pohl H, von Reumont BM, Schutte K, Sekiya K, Shimizu S, Slipinski A, Stamatakis A, Song W, Su X, Szucsich NU, Tan M, Tan X, Tang M, Tang J, Timelthaler G, Tomizuka S, Trautwein M, Tong X, Uchifune T, Walzl MG, Wiegmann BM, Wilbrandt J, Wipfler B, Wong TKF, Wu Q, Wu G, Xie Y, Yang S, Yang Q, Yeates DK, Yoshizawa K, Zhang Q, Zhang R, Zhang W, Zhang Y, Zhao J, Zhou C, Zhou L, Ziesmann T, Zou S, Li Y, Xu X, Zhang Y, Yang H, Wang J, Wang J, Kjer KM, Zhou X. Phylogenomics resolves the timing and pattern of insect evolution. Science. 2014; 346(6210):763–7. doi:10.1126/science.1257570. Accessed 15 Nov 2014.

[15] Struck TH, Paul C, Hill N, Hartmann S, Hösel C, Kube M, Lieb B, Meyer A, Tiedemann R, Purschke G, Bleidorn C. Phylogenomic analyses unravel annelid evolution. Nature. 2011; 471(7336):95–8. doi:10.1038/nature09864. Accessed 21 Jul 2016. 10.1038/nature09864 21368831

[16] Kvist S, Siddall ME. Phylogenomics of Annelida revisited: A cladistic approach using genome-wide expressed sequence tag data mining and examining the effects of missing data. Cladistics. 2013; 29(4):435–48. doi:10.1111/cla.12015. Accessed 9 Sept 2015. 10.1111/cla.12015 34798767

[17] Slater G, Birney E. Automated generation of heuristics for biological sequence comparison. Bmc Bioinformatics. 2005; 6(1):31. doi:10.1186/1471-2105-6-31. 10.1186/1471-2105-6-31 PMC553969 15713233

[18] Honeybee Genome Sequencing Consortium. Insights into social insects from the genome of the honeybee Apis mellifera. Nature. 2006; 443(7114):931–49. doi:10.1038/nature05260. 10.1038/nature05260 PMC2048586 17073008

[19] Bonasio R, Zhang G, Ye C, Mutti NS, Fang X, Qin N, Donahue G, Yang P, Li Q, Li C, Zhang P, Huang Z, Berger SL, Reinberg D, Wang J, Liebig J (2010). Genomic comparison of the ants Camponotus floridanus and Harpegnathos saltator. Science.

[20] Waterhouse RM, Zdobnov EM, Tegenfeldt F, Li J, Kriventseva EV (2011). OrthoDB: The hierarchical catalog of eukaryotic orthologs in 2011. Nucleic Acids Res.

[21] Werren JH, Richards S, Desjardins CA, Niehuis O, Gadau J, Colbourne JK, Beukeboom LW, Desplan C, Elsik CG, Grimmelikhuijzen CJP, Kitts P, Lynch JA, Murphy T, Oliveira DCSG, Smith CD, v d Zande L, Worley KC, Zdobnov EM, Aerts M, Albert S, Anaya VH, Anzola JM, Barchuk AR, Behura SK, Bera AN, Berenbaum MR, Bertossa RC, Bitondi MMG, Bordenstein SR, Bork P, Bornberg-Bauer E, Brunain M, Cazzamali G, Chaboub L, Chacko J, Chavez D, Childers CP, Choi JH, Clark ME, Claudianos C, Clinton RA, Cree AG, Cristino AS, Dang PM, Darby AC, de Graaf DC, Devreese B, Dinh HH, Edwards R, Elango N, Elhaik E, Ermolaeva O, Evans JD, Foret S, Fowler GR, Gerlach D, Gibson JD, Gilbert DG, Graur D, Grunder S, Hagen DE, Han Y, Hauser F, Hultmark D, Hunter HC, Hurst GDD, Jhangian SN, Jiang H, Johnson RM, Jones AK, Junier T, Kadowaki T, Kamping A, Kapustin Y, Kechavarzi B, Kim J, Kim J, Kiryutin B, Koevoets T, Kovar CL, Kriventseva EV, Kucharski R, Lee H, Lee SL, Lees K, Lewis LR, Loehlin DW, Logsdon JM, Lopez JA, Lozado RJ, Maglott D, Maleszka R, Mayampurath A, Mazur DJ, McClure MA, Moore AD, Morgan MB, Muller J, Munoz-Torres MC, Muzny DM, Nazareth LV, Neupert S, Nguyen NB, Nunes FMF, Oakeshott JG, Okwuonu GO, Pannebakker BA, Pejaver VR, Peng Z, Pratt SC, Predel R, Pu LL, Ranson H, Raychoudhury R, Rechtsteiner A, Reid JG, Riddle M, Romero-Severson J, Rosenberg M, Sackton TB, Sattelle DB, Schluns H, Schmitt T, Schneider M, Schuler A, Schurko AM, Shuker DM, Simoes ZLP, Sinha S, Smith Z, Souvorov A, Springauf A, Stafflinger E, Stage DE, Stanke M, Tanaka Y, Telschow A, Trent C, Vattathil S, Viljakainen L, Wanner KW, Waterhouse RM, Whitfield JB, Wilkes TE, Williamson M, Willis JH, Wolschin F, Wyder S, Yamada T, Yi SV, Zecher CN, Zhang L, Gibbs RA, The Nasonia Genome Working Group (2010). Functional and evolutionary insights from the genomes of three parasitoid Nasonia species. Science.

[22] Richards S, Gibbs RA, Weinstock GM, Brown SJ, Denell R, Beeman RW, Gibbs R, Bucher G, Friedrich M, Grimmelikhuijzen CJP, Klingler M, Lorenzen M, Roth S, Schröder R, Tautz D, Zdobnov EM, Muzny D, Attaway T, Bell S, Buhay CJ, Chandrabose MN, Chavez D, Clerk-Blankenburg KP, Cree A, Dao M, Davis C, Chacko J, Dinh H, Dugan-Rocha S, Fowler G, Garner TT, Garnes J, Gnirke A, Hawes A, Hernandez J, Hines S, Holder M, Hume J, Jhangiani SN, Joshi V, Khan ZM, Jackson L, Kovar C, Kowis A, Lee S, Lewis LR, Margolis J, Morgan M, Nazareth LV, Nguyen N, Okwuonu G, Parker D, Ruiz SJ, Santibanez J, Savard J, Scherer SE, Schneider B, Sodergren E, Vattahil S, Villasana D, White CS, Wright R, Park Y, Lord J, Oppert B, Brown S, Wang L, Weinstock G, Liu Y, Worley K, Elsik CG, Reese JT, Elhaik E, Landan G, Graur D, Arensburger P, Atkinson P, Beidler J, Demuth JP, Drury DW, Du YZ, Fujiwara H, Maselli V, Osanai M, Robertson HM, Tu Z, Wang J-j, Wang S, Song H, Zhang L, Werner D, Stanke M, Morgenstern B, Solovyev V, Kosarev P, Brown G, Chen HC, Ermolaeva O, Hlavina W, Kapustin Y, Kiryutin B, Kitts P, Maglott D, Pruitt K, Sapojnikov V, Souvorov A, Mackey AJ, Waterhouse RM, Wyder S, Kriventseva EV, Kadowaki T, Bork P, Aranda M, Bao R, Beermann A, Berns N, Bolognesi R, Bonneton F, Bopp D, Butts T, Chaumot A, Denell RE, Ferrier DEK, Gordon CM, Jindra M, Lan Q, Lattorff HMG, Laudet V, von Levetsow C, Liu Z, Lutz R, Lynch JA, da Fonseca RN, Posnien N, Reuter R, Schinko JB, Schmitt C, Schoppmeier M, Shippy TD, Simonnet F, Marques-Souza H, Tomoyasu Y, Trauner J, der Zee MV, Vervoort M, Wittkopp N, Wimmer EA, Yang X, Jones AK, Sattelle DB, Ebert PR, Nelson D, Scott JG, Muthukrishnan S, Kramer KJ, Arakane Y, Zhu Q, Hogenkamp D, Dixit R, Jiang H, Zou Z, Marshall J, Elpidina E, Vinokurov K, Oppert C, Evans J, Lu Z, Zhao P, Sumathipala N, Altincicek B, Vilcinskas A, Williams M, Hultmark D, Hetru C, Hauser F, Cazzamali G, Williamson M, Li B, Tanaka Y, Predel R, Neupert S, Schachtner J, Verleyen P, Raible F, Walden KKO, Angeli S, Forêt S, Schuetz S, Maleszka R, Miller SC, Grossmann D (2008). The genome of the model beetle and pest Tribolium castaneum. Nature.

[23] Suen G, Teiling C, Li L, Holt C, Abouheif E, Bornberg-Bauer E, Bouffard P, Caldera EJ, Cash E, Cavanaugh A, Denas O, Elhaik E, Favé M-J, Gadau J, Gibson JD, Graur D, Grubbs KJ, Hagen DE, Harkins TT, Helmkampf M, Hu H, Johnson BR, Kim J, Marsh SE, Moeller JA, Muñoz-Torres MC, Murphy MC, Naughton MC, Nigam S, Overson R, Rajakumar R, Reese JT, Scott JJ, Smith CR, Tao S, Tsutsui ND, Viljakainen L, Wissler L, Yandell MD, Zimmer F, Taylor J, Slater SC, Clifton SW, Warren WC, Elsik CG, Smith CD, Weinstock GM, Gerardo NM, Currie CR. The genome gequence of the leaf-cutter ant Atta cephalotes reveals insights into Its obligate symbiotic lifestyle. PLoS Genet. 2011; 7(2):1002007. doi:10.1371/journal.pgen.1002007. Accessed 10 Aug 2015. 10.1371/journal.pgen.1002007 PMC3037820 21347285

[24] Struck TH, Wey-Fabrizius AR, Golombek A, Hering L, Weigert A, Bleidorn C, Klebow S, Iakovenko N, Hausdorf B, Petersen M, Kuck P, Herlyn H, Hankeln T (2014). Platyzoan paraphyly based on phylogenomic data supports a noncoelomate ancestry of Spiralia. Mol Biol Evol.

[25] Jarvis ED, Mirarab S, Aberer AJ, Li B, Houde P, Li C, Ho SYW, Faircloth BC, Nabholz B, Howard JT, Suh A, Weber CC, da Fonseca RR, Li J, Zhang F, Li H, Zhou L, Narula N, Liu L, Ganapathy G, Boussau B, Bayzid MS, Zavidovych V, Subramanian S, Gabaldon T, Capella-Gutierrez S, Huerta-Cepas J, Rekepalli B, Munch K, Schierup M, Lindow B, Warren WC, Ray D, Green RE, Bruford MW, Zhan X, Dixon A, Li S, Li N, Huang Y, Derryberry EP, Bertelsen MF, Sheldon FH, Brumfield RT, Mello CV, Lovell PV, Wirthlin M, Schneider MPC, Prosdocimi F, Samaniego JA, Velazquez AMV, Alfaro-Nunez A, Campos PF, Petersen B, Sicheritz-Ponten T, Pas A, Bailey T, Scofield P, Bunce M, Lambert DM, Zhou Q, Perelman P, Driskell AC, Shapiro B, Xiong Z, Zeng Y, Liu S, Li Z, Liu B, Wu K, Xiao J, Yinqi X, Zheng Q, Zhang Y, Yang H, Wang J, Smeds L, Rheindt FE, Braun M, Fjeldsa J, Orlando L, Barker FK, Jonsson KA, Johnson W, Koepfli KP, O’Brien S, Haussler D, Ryder OA, Rahbek C, Willerslev E, Graves GR, Glenn TC, McCormack J, Burt D, Ellegren H, Alstrom P, Edwards SV, Stamatakis A, Mindell DP, Cracraft J, Braun EL, Warnow T, Jun W, Gilbert MTP, Zhang G. Whole-genome analyses resolve early branches in the tree of life of modern birds. Science. 2014; 346(6215):1320–31. doi:10.1126/science.1253451. Accessed 8 Oct 2015. 10.1126/science.1253451 PMC4405904 25504713

[26] Hipp RD, Kennedy D, Mistachkin J. SQLite. 2016. https://www.sqlite.org. Accessed 2015-11-03.

[27] Capella-Gutierrez S, Kauff F, Gabaldón T. A phylogenomics approach for selecting robust sets of phylogenetic markers. Nucl. Acids Res. 2014; 071. doi:10.1093/nar/gku071. Accessed 18 Oct 2016. 10.1093/nar/gku071 PMC3985644 24476915

[28] Denton JF, Lugo-Martinez J, Tucker AE, Schrider DR, Warren WC, Hahn MW (2014). Extensive error in the number of genes inferred from draft genome assemblies. PLoS Comput Biol.

[29] Eddy SR (2011). Accelerated Profile HMM Searches. PLoS Comput Biol.

[30] Rognes T (2011). Faster Smith-Waterman database searches with inter-sequence SIMD parallelisation. BMC Bioinformatics.

[31] Mayer C, Sann M, Donath A, Meixner M, Podsiadlowski L, Peters RS, Petersen M, Meusemann K, Liere K, Wägele JW, Misof B, Bleidorn C, Ohl M, Niehuis O. BaitFisher: A Software Package for Multispecies Target DNA Enrichment Probe Design. Mol Biol Evol. 2016; 33(7):1875–86. doi:10.1093/molbev/msw056. Accessed 11 Jan 2017. 10.1093/molbev/msw056 27009209

