# Supplementary material for: Orthograph: a versatile tool for mapping coding nucleotide sequences to clusters of orthologous genes
Source: BMC Bioinformatics. 2017 Feb 16;18:111. doi: 10.1186/s12859-017-1529-8 (PMC5312442; doi:10.1186/s12859-017-1529-8)
Supplement: Additional file 1 — Supplemental methods and data tables. Figure S1. Alignment regions in Orthograph; Figure S2. ORF extension criteria; Figure S3. Orthograph runtime is significantly correlated to total transcriptome assembly length; Figure S4. Speedup plot for multi-threaded analysis; Figure S5. Example multiple sequence alignment of an OG to demonstrate a possible assignment of a transcript to the “wrong” OG. (PDF 1020 kb) [file 12859_2017_1529_MOESM1_ESM.pdf]

Orthograph: A versatile tool for mapping coding nucleotide sequences to clusters of orthologous genes

Supplemental Material

Methods

Apoid wasp transcriptomes

We *de novo* sequenced whole body transcript libraries of 24 apoid wasp species in the context of the international 1KITE project (Table S1). Adult wasps were collected via hand-netting and immediately preserved in RNAlater. RNA extraction, cDNA synthesis, and sequencing library preparation followed the methodology outlined by [1]. Briefly, RNA was extracted using a standard phenol/guanidine isothiocyanate-based extraction method and tested for quality before processing for library construction. cDNA libraries were constructed by shearing and amplification of mRNA that was isolated using magnetic beads. A random hexamer primer was added and the double-stranded cDNA then underwent end-repair, a single 'A' base addition and adapter ligation. Library size selection was performed by gel electrophoresis and excision of the 250 $\pm$ 20 bp band. The product was indexed and PCR amplified to obtain paired-end cDNA. After cDNA fragment size verification, the cDNA libraries were sequenced on an Illumina HiSeq2000 platform following standard protocols. For each library, roughly 2.5 Gbp of raw data was sequenced with 150 bp paired-end reads. After filtering steps to ensure high quality raw data libraries, transcripts were assembled with SOAPdenovo-trans-31kmer v1.01 [2] with moderately strict parameters (−e3). The assembled transcript

libraries were finally screened for vector and adapter contamination using a local VecScreen installation (http://www.ncbi.nlm.nih.gov/tools/vecscreen) and the UniVec database build 7.0 (http://www.ncbi.nlm.nih.gov/tools/vecscreen/univec). Assembled transcripts at least 200 bp in length were checked for cross-contamination with reads from samples sequenced on the same Illumina lane. In brief, BLAST hits with lengths > 179 and identity of at least 98% were compared for their k-mer coverage values (as computed during assembly with SOAPdenovo-trans). From a cluster of highly similar sequences only the sequence with highest k-mer coverage was kept, and only if the coverage was at least 2x higher than the second best, otherwise all were discarded. The remaining contigs were submitted to the NCBI Transcriptome Shotgun Assembly (TSA) database, where they were again screened for potential contaminants from vector nucleotide sequences as well as for sequences that might originate from non-target species contamination.

Sequencing data were deposited at the Sequence Read Archive (SRA) and the Transcriptome Shotgun Assembly (TSA) database of NCBI GenBank (accession numbers see Additional file 2) and are available at NCBI via the Umbrella BioProject ID PRJNA183205 ("The 1KITE project: evolution of insects").

Orthograph

Orthograph dependencies

Orthograph requires the software packages HMMER3 [3], NCBI BLAST+ [4], MAFFT [5], and Exonerate [6] as well as either MySQL or SQLite (for specific version see Table S1).

Ortholog reference set

The user must provide Orthograph with a set of reference OGs to which transcripts are mapped. For this purpose, Orthograph requires the amino acid and corresponding nucleotide sequences of all protein-coding genes in the user-selected reference OGS. It additionally needs information about which genes in these genomes are orthologous. Information on orthology relations of genes in the reference genomes (*i.e.*, what genes form OGs) can be obtained from databases such as OrthoDB (http://orthodb.org), InParanoid (http://inparanoid.sbc.su.se), OrthoMCL DB (http://orthomcl.org), and OMA (http://omabrowser.org). Alternatively, a reference ortholog set has to be inferred using an orthology prediction approach that works on fully sequenced genomes, such as the respective tools for the databases (OrthoDB [7],

OrthoMCL [8], InParanoid [9], OMA [10]) or the pipeline OrthoFinder [11]. To construct a reference set of OGs for identifying orthologous *de novo*-sequenced transcripts of 24 apoid wasps (see below), we exploited OrthoDB 5 [12], a database that delineates orthologs among published genomes using a graph-based clustering strategy. For the analysis of the 24 *de novo*-sequenced transcript libraries of apoid wasps, we used a reference ortholog set that contains OGs from six species of Hymenoptera: *Acromyrmex echinatior*, *Apis mellifera*, *Camponotus floridanus*, *Harpegnathos saltator*, *Linepithema humile*, and *Nasonia vitripennis*. The OGS versions and download URLs are listed in Table S3. These taxa were selected because a) their genomes are well sequenced, fully annotated, published, and publicly available, and b) they represent major lineages of Hymenoptera comparatively closely related to apoid wasps. The hierarchical level for clustering orthologous genes in the OrthoDB query was set to the node Apocrita (*N. vitripennis*/rest of Hymenoptera). We requested genes in the above six reference species to be always present in single copy. Given these settings, OrthoDB 5 identified 5,561 OGs fulfilling these criteria. The resulting OrthoDB table was subsequently filtered to only contain information about the selected taxa. Since Orthograph needs to relate identifiers in the OrthoDB table to sequences in the OGS, headers in the OGS files were modified so that they match the header naming scheme in the OrthoDB table. For testing the functionality and performance of Orthograph, we used a different set (see below).

Scan for candidate transcripts using profile hidden Markov models

Orthograph creates a multiple sequence alignment (MSA) from the individual amino acid sequences that are part of a given OG using MAFFT L-INS-I [5]. From each of the resulting MSAs, Orthograph constructs a profile hidden Markov model (pHMM) using HMMER3 with default parameters, resulting in one pHMM per OG. Orthograph uses these pHMMs to search the transcript library (or any other pool of coding sequences that can also include short non-coding sequence sections, such as introns) for candidate orthologs on amino acid level in all six possible reading frames. Orthograph allows the user to specify an alternative genetic code translation table when dealing with species that use a different genetic code. All search results are stored in a relational database for later evaluation; note that no orthology delineation is performed at this point. As relational database management system, the user can chose between MySQL and SQLite. The first is a reasonable choice when running in a network environment with one computer acting as a database server; the latter when running Orthograph on a HPC cluster.

Establishing BRH criterion using BLAST+

A BLAST database is generated from all amino acid sequences of all reference proteomes. Orthograph uses the predicted amino acid sequence section of a candidate transcript (or other coding sequences) that returned a match during the pHMM search as query for a search against the above reference proteome database using protein BLAST of the NCBI BLAST+ program suite. All retrieved search results are subsequently stored in the database for later evaluation. Note that in contrast to the algorithm in HaMStR, Orthograph attempts no orthology delineation at this point.

Extension of clusters of orthologous genes

Orthograph retrieves the results from all pHMM searches from the database sorted by descending alignment bit score. Sorting by bit score increases the likelihood of retrieving the biologically most relevant hit by using sequence similarity as a criterion for putative sequence homology. For each candidate transcript, the search results are tested for reciprocity: if the subsequent reverse BLAST search using the candidate transcript as query matches a target sequence from the OGS that is part of the OG that formed the basis for this particular pHMM, the BRH criterion is fulfilled. In this case, an ortholog relationship between the target transcript section and the OG is assumed and the target transcript section is assigned to the OG unless it overlaps with a previous assignment. If it overlaps with a previous assignment, *i.e.* two different sequences fulfilling the BRH criterion on overlapping regions of the OG, a paralogous relationship is assumed and the transcript section is recorded accordingly. To avoid protein domain walking, Orthograph does not consider transcript sections of fewer than 30 amino acids in length for forther processing. This cutoff can be changed by the user, if necessary.

Frameshift-corrected ORF inference

To infer ORFs and to correct for frameshift errors, which may be present in NGS products, Orthograph employs the alignment program Exonerate [6]. It is used to compute a pairwise alignment of the amino acid sequence of the most similar reference taxon and the orthologous transcript section on nucleotide level to infer the corresponding coding DNA sequence. As a result, Orthograph provides corresponding amino acid and nucleotide sequences for the orthologous transcripts. Orthograph can extend the ORF beyond the pHMM alignment coordinates by inferring ORFs from the entire transcript sequence. More than 50% (default

value that can be changed by the user) of the resulting ORF must be part of the sequence region for which orthology has been inferred (Figure S2). This is done to obtain a longer ORF while retaining orthology information for the majority of its length.

Reanalysis of publicly available data

Sensitivity and accuracy when searching for single-copy orthologs

From the OrthoDB 7 database [13], a set of OGs was obtained for four species of Hymenoptera and an outgroup beetle. The hierarchical level was set to the split (Hymenoptera/rest of Holometabola) and we requested that genes in *A. mellifera*, *C. floridanus*, *H. saltator*, *N. vitripennis*, and *T. castaneum* occur in single-copy, while copy number in all other taxa was left unspecified. This query returned 4,625 OGs. The resulting table was filtered to contain only entries from the above five species. This table was re-filtered twice to obtain two different ortholog sets: one that was missing entries from *A. mellifera*, and one that excluded entries from *H. saltator*. Note that we included only the longest isoform per gene from the OGS libraries, irrespective of the species. The sets were imported into Orthograph. We ran the analysis using default parameters. Evaluation was performed using custom-made Bash scripts.

Identification of splice variants or isoforms

To identify splice variants or isoforms, we used the ortholog set derived from five reference species with 4,625 OGs from the analysis for testing Orthograph performance when searching for single-copy orthologs. We included sequences from all five species in the set. Additionally, we downloaded the *C. floridanus* OGS transcripts from the Hymenoptera Genome Database [14]. The sequence headers were reformatted to match the format used in the OrthoDB table. The ortholog set was imported in the Orthograph database, and we ran the analysis using default parameters. The results were evaluated using custom-made Bash scripts.

Identification of inparalogs

We complemented the ortholog set from the analysis for testing Orthograph sensitivity and accuracy when searching for single-copy orthologs with amino acid sequences from *A. cephalotes* by a modified query to OrthoDB 7, demanding presence, but without copy-number restriction for genes from *A. cephalotes*. We obtained the OGS of *A. cephalotes*, version 1.2, from http://www.hymenopteragenome.org/atta/?q=genome_consortium_datasets [15].

Phylogenetic split as well as copy-number restrictions for the other taxa were kept as described above. This query returned 301 OGs. The resulting table was filtered to contain only entries from the six selected species *A. mellifera*, *C. floridanus*, *H. saltator*, *N. vitripennis*, *T. castaneum*, and *A. cephalotes*. The set was imported into the Orthograph database, and we ran the analysis using default parameters. The results were evaluated using custom-made Bash and Perl scripts.

Non-redundant mapping of transcripts

The dataset from Struck *et al.* [16] was obtained from the Dryad database (http://datadryad.org/bitstream/handle/10255/dryad.62820/Struck_Platyzoa2014.tgz). The ortholog set used by Struck *et al.* [16] was obtained from the HaMStR website at http://deep-phylogeny.org/hamstr/download/datasets/hmmer3/lophotrochozoa_hmmer3.tar.gz. The reference OGSs provided in the online material from *Struck et al.* [16] were reformatted and imported into Orthograph. We ran the analysis with parameters that closely resemble the settings in HaMStR used by *Struck et al.* [16]. Evaluation of the annotation result was performed using custom-made Bash and Perl scripts.

Computational performance

We tested the computational performance of Orthograph by running analyses on a workstation computer with an Intel Core i7 quad-core processor (3.4 GHz) and 8 GB of RAM. We used the same set of 5,561 single-copy orthologs that was used by Mayer et al. [17]. For testing the multi-threaded performance, we used a HPC machine with two 6-core Intel Xeon processors (2.67 GHz) capable of running 24 parallel threads total. Orthograph was run on a medium-sized transcriptome of the 24 apoid wasp transcriptomes (*Chalybion californicum*, with 34 Mbp) with default settings and using 1 to 16 parallel threads.

Table S1: Software packages required by Orthograph. It has been developed and tested with these versions. Older versions are not supported.

Package	Version	Download from
Perl	5.14	http://www.perl.org
SQLite	3.8.2	http://sqlite.org/download.html
MySQL	5.6.17	http://dev.mysql.com/downloads/mysql/
MAFFT	7.023b	http://mafft.cbrc.jp/alignment/software/
HMMer	3.1b1	http://hmmer.janelia.org/software/
NCBI BLAST+	2.2.28+	ftp://ftp.ncbi.nlm.nih.gov/blast/executables/blast+/LATEST/
Exonerate	2.2.0	http://www.ebi.ac.uk/~guy/exonerate/

Table S2: Species for which 1KITE transcriptomes were analyzed.

Order	Family	Subfamily	Genus	Species
Hymenoptera	Crabronidae	Bembicinae	Alyssontini	*Alysson spinosus*
Hymenoptera	Crabronidae	Bembicinae	Bembicini	*Bembix rostrata*
Hymenoptera	Crabronidae	Bembicinae	Bembicini	*Gorytes laticinctus*
Hymenoptera	Crabronidae	Bembicinae	Bembicini	*Harpactus elegans*
Hymenoptera	Crabronidae	Bembicinae	Bembicini	*Sphecius convallis*
Hymenoptera	Crabronidae	Bembicinae	Bembicini	*Stizoides tridentatus*
Hymenoptera	Crabronidae	Bembicinae	Nyssonini	*Nysson niger*
Hymenoptera	Crabronidae	Crabroninae	Crabronini	*Crabro peltarius*
Hymenoptera	Crabronidae	Crabroninae	Crabronini	*Crossocerus quadrimaculatus*
Hymenoptera	Crabronidae	Crabroninae	Larrini	*Tachysphex fulvitarsis*
Hymenoptera	Crabronidae	Crabroninae	Oxybelini	*Oxybelus bipunctatus*
Hymenoptera	Crabronidae	Crabroninae	Trypoxylini	*Trypoxylon figulus*
Hymenoptera	Crabronidae	Dinetinae	-	*Dinetus pictus*
Hymenoptera	Crabronidae	Pemphredoninae	Pemphredonini	*Diodontus minutus*
Hymenoptera	Crabronidae	Pemphredoninae	Pemphredonini	*Pemphredon lugens*
Hymenoptera	Crabronidae	Pemphredoninae	Psenini	*Psenulus fuscipennis*
Hymenoptera	Crabronidae	Philanthinae	Cercerini	*Cerceris arenaria*
Hymenoptera	Crabronidae	Philanthinae	Philanthini	*Philanthus triangulum*
Hymenoptera	Sphecidae	Ammophilinae	-	*Podalonia hirsuta*
Hymenoptera	Sphecidae	Sceliphrinae	Sceliphrini	*Chalybion californicum*
Hymenoptera	Sphecidae	Sceliphrinae	Sceliphrini	*Sceliphron curvatum*
Hymenoptera	Sphecidae	Sphecinae	Prionychini	*Prionyx kirbii*
Hymenoptera	Sphecidae	Sphecinae	Sphecini	*Isodontia mexicana*
Hymenoptera	Sphecidae	Sphecinae	Sphecini	*Sphex funerarius*

Table S3: Official gene sets for the reference ortholog set generation.

Species	Version	URL	Citation
Acromyrmex echinatior	1.2	http://hymenopteragenome.org/acromyrmex/?q=genome_consortium_datasets	[18]
Apis mellifera	1.1	http://hymenopteragenome.org/beebase/?q=download_sequence	[19]
Atta cephalotes	1.2	http://hymenopteragenome.org/atta/?q=genome_consortium_datasets	[15]
Camponotus floridanus	3.3	http://hymenopteragenome.org/camponotus/?q=genome_consortium_datasets	[20]
Harpegnathos saltator	3.3	http://hymenopteragenome.org/harpegnathos/?q=genome_consortium_datasets	[20]
Linepithema humile	1.2	http://hymenopteragenome.org/linepithema/?q=genome_consortium_datasets	[21]
Nasonia vitripennis	1.2	http://hymenopteragenome.org/nasonia/?q=sequencing_and_analysis_consortium_datasets	[22]
Tribolium castaneum	3.0	http://beetlebase.org/?q=download_settings	[23]

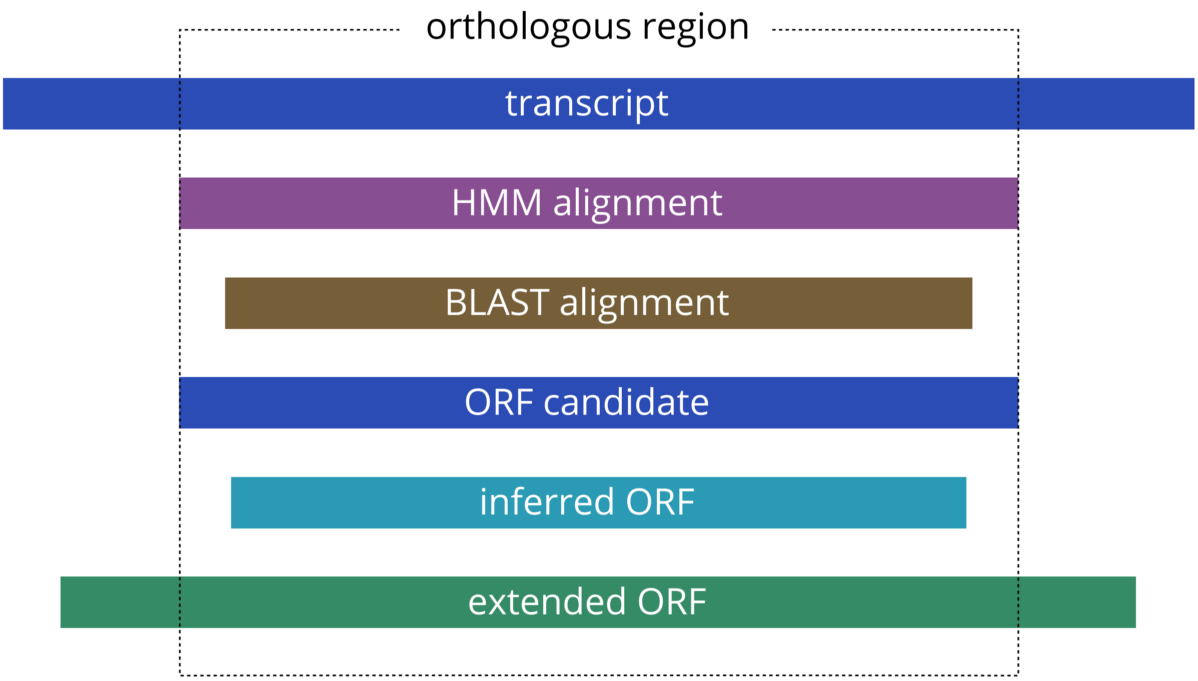

Figure S1: Alignment regions in Orthograph. On the transcript, there is a candidate ortholog region that was identified using a HMM alignment. The reverse search result using BLAST confirms orthology for the candidate region. For ORF inference, the transcript subsequence that was identified as putatively orthologous using the HMM search is used. The resulting ORF may then be extended by using the entire transcript sequence, resulting in ORF coordinates that exceed the orthologous region.

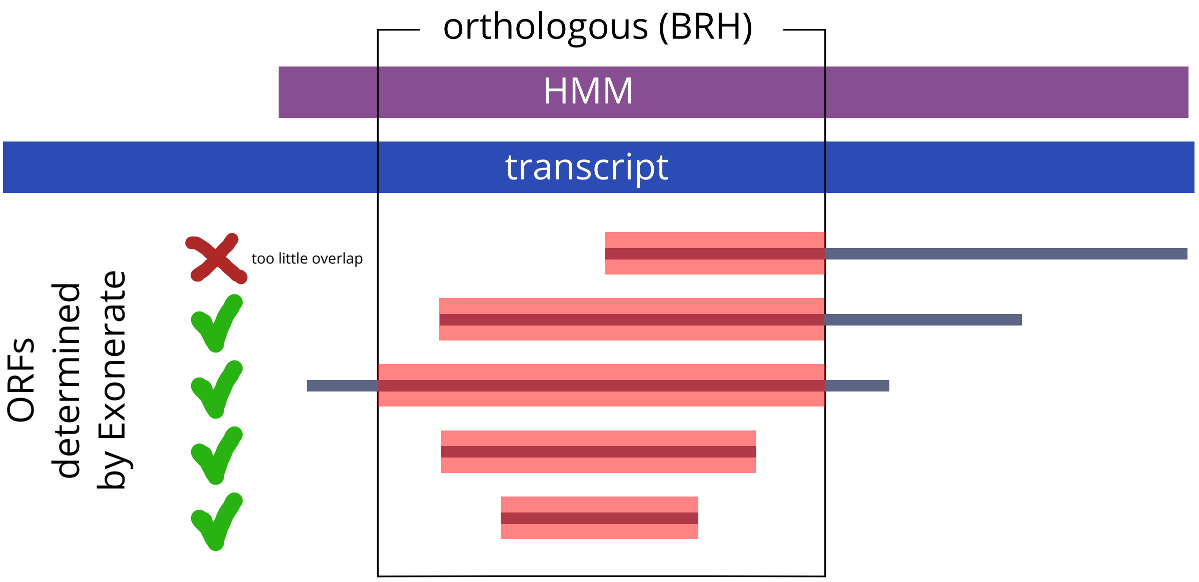

Figure S2: ORF extension criteria in Orthograph. Inferred ORFs that do not overlap at least 50 % of the orthologous region are discarded due to insufficient confidence in orthology status. As long as the majority of the ORF length is inside the orthologous region on the transcript, ORFs are accepted.

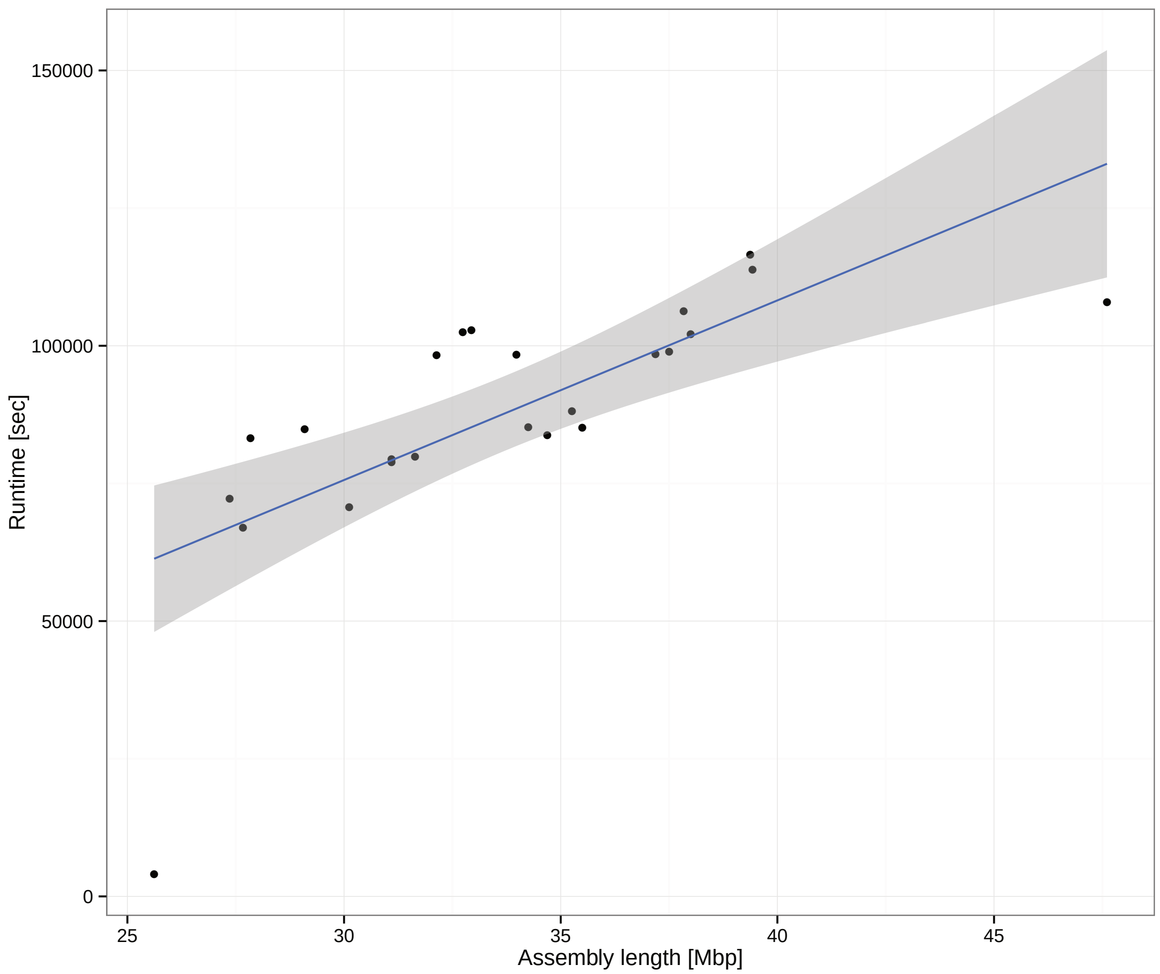

Figure S3: Orthograph runtime is significantly correlated to total transcriptome assembly length (Spearman rank correlation, $S = 326$, $p \ll 0.001$) when running with a single thread. Dots indicate measurements for individual transcriptome assemblies. Blue line: linear regression model; gray area: confidence interval.

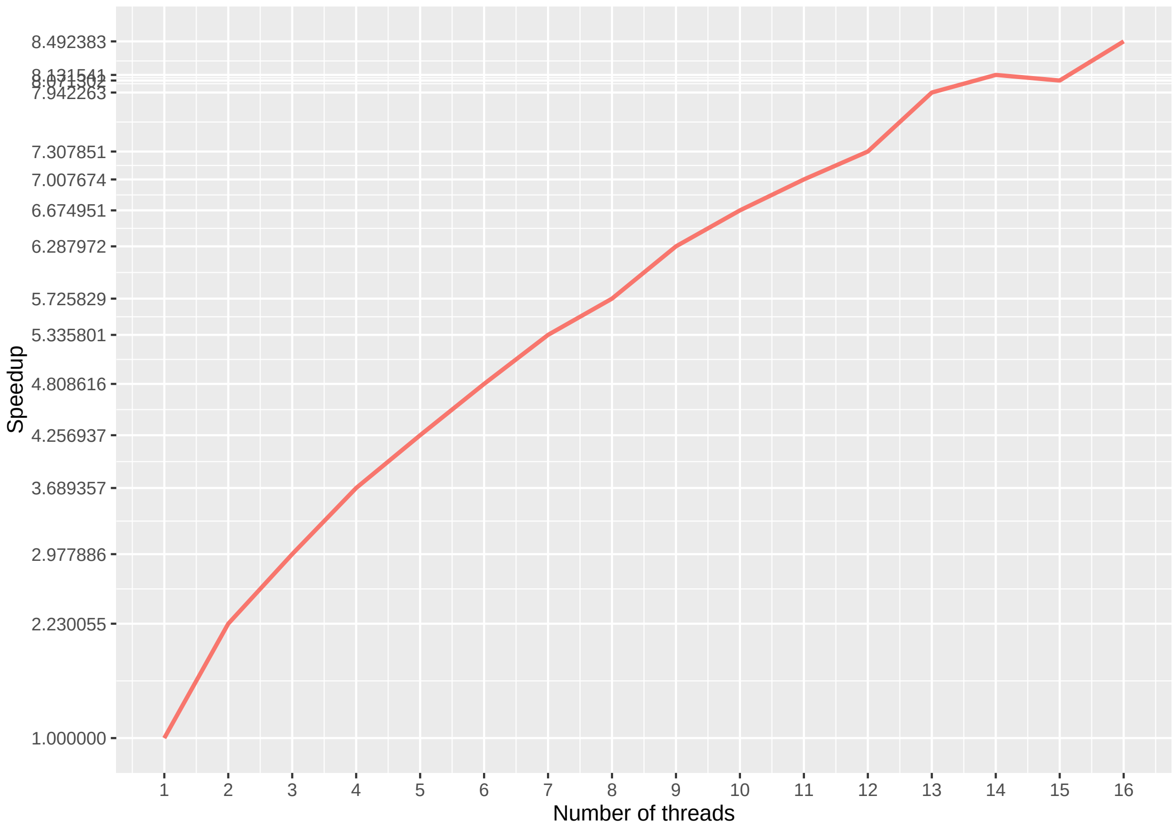

Figure S4: Orthograph profits from multiple CPU threads. The x axis shows the number of CPU threads; the y axis shows the relative speedup compared to single-threaded performance on a transcriptome assembly of 34 Mbp. Using 16 threads reduces Orthograph runtime to 11.7 % of single-threaded runtime.

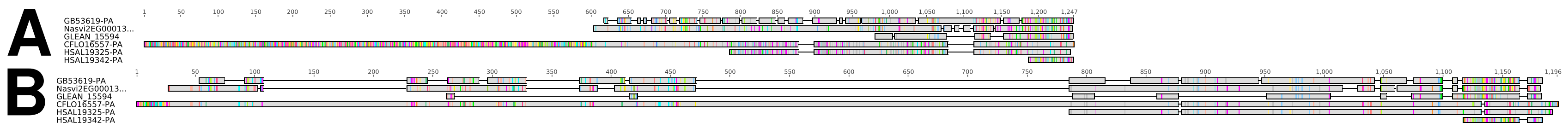

Figure S5: Multiple sequence alignment (MSA) of an ortholog group (OG) as an examplary assignment of a gene from the *H. saltator* reference gene set (RGS) to the "wrong" OG. A: Alignment using the ClustalW algorithm [24]; B: Alignment using the MUSCLE algorithm [25]. According to OrthoDB, the protein HSAL19342-PA belongs to the OG with the ID EOG7KHN7Q. Orthograph, however, identified the protein HSAL19325-PA as orthologous to this OG due to a high similarity to one of the proteins in the OG (404 amino acids alignment overlap, 64.6 % identical sites). The sequence HSAL19325-PA has been added to the MSA to demonstrate that it is in large parts more similar to a sequence from *C. floridanus* (CFLO16557-PA) and therefore yields a higher alignment bit score than the correct – according to OrthoDB – ortholog HSAL19342-PA. In contrast, the protein that is recorded in OrthoDB as part of the OG is shorter and displays little similarity (61 amino acids alignment overlap, 31.1 % identical sites). This leads to a higher bit score in the reverse search for the longer and more similar – but not orthologous according to OrthoDB – sequence. In turn, the BRH criterion for the correct, but shorter ortholog (according to OrthoDB) was not fulfilled. This demonstrates that using different alignment algorithms can impede successful orthology assignment. Grey areas indicate conserved regions, colored bars indicate sequence-specific different amino acid positions. Graphic created using Geneious v7.1 (http://www.geneious.com).

References

[1] B. Misof, S. Liu, K. Meusemann, R. S. Peters, A. Donath, C. Mayer, P. B. Frandsen, J. Ware, T. Flouri, R. G. Beutel, O. Niehuis, M. Petersen, F. Izquierdo-Carrasco, T. Wappler, J. Rust, A. J. Aberer, U. Aspock, H. Aspock, D. Bartel, A. Blanke, S. Berger, A. Bohm, T. R. Buckley, B. Calcott, J. Chen, F. Friedrich, M. Fukui, M. Fujita, C. Greve, P. Grobe, S. Gu, Y. Huang, L. S. Jermiin, A. Y. Kawahara, L. Krogmann, M. Kubiak, R. Lanfear, H. Letsch, Y. Li, Z. Li, J. Li, H. Lu, R. Machida, Y. Mashimo, P. Kapli, D. D. McKenna, G. Meng, Y. Nakagaki, J. L. Navarrete-Heredia, M. Ott, Y. Ou, G. Pass, L. Podsiadlowski, H. Pohl, B. M. von Reumont, K. Schutte, K. Sekiya, S. Shimizu, A. Slipinski, A. Stamatakis, W. Song, X. Su, N. U. Szucsich, M. Tan, X. Tan, M. Tang, J. Tang, G. Timelthaler, S. Tomizuka, M. Trautwein, X. Tong, T. Uchifune, M. G. Walzl, B. M. Wiegmann, J. Wilbrandt, B. Wipfler, T. K. F. Wong, Q. Wu, G. Wu, Y. Xie, S. Yang, Q. Yang, D. K. Yeates, K. Yoshizawa, Q. Zhang, R. Zhang, W. Zhang, Y. Zhang, J. Zhao, C. Zhou, L. Zhou, T. Ziesmann, S. Zou, Y. Li, X. Xu, Y. Zhang, H. Yang, J. Wang, J. Wang, K. M. Kjer, and X. Zhou, "Phylogenomics resolves the timing and pattern of insect evolution," *Science*, vol. 346, pp. 763–767, Nov. 2014.

[2] Y. Xie, G. Wu, J. Tang, R. Luo, J. Patterson, S. Liu, W. Huang, G. He, S. Gu, S. Li, X. Zhou, T.-W. Lam, Y. Li, X. Xu, G. K.-S. Wong, and J. Wang, "SOAPdenovo-Trans: De novo transcriptome assembly with short RNA-Seq reads," *Bioinformatics*, vol. 30, pp. 1660–1666, June 2014.

[3] S. R. Eddy, "Accelerated Profile HMM Searches," *PLoS Computational Biology*, vol. 7, p. e1002195, Oct. 2011.

[4] C. Camacho, G. Coulouris, V. Avagyan, N. Ma, J. Papadopoulos, K. Bealer, and T. L. Madden, "BLAST+: Architecture and applications," *BMC Bioinformatics*, vol. 10, no. 1, p. 421, 2009.

[5] K. Katoh and D. M. Standley, "MAFFT Multiple sequence alignment software version 7: Improvements in performance and usability," *Mol Biol Evol*, vol. 30, pp. 772–780, Jan. 2013.

[6] G. Slater and E. Birney, "Automated generation of heuristics for biological sequence comparison," *Bmc Bioinformatics*, vol. 6, no. 1, p. 31, 2005.

[7] E. V. Kriventseva, F. Tegenfeldt, T. J. Petty, R. M. Waterhouse, F. A. Simao, I. A. Pozdnyakov, P. Ioannidis, and E. M. Zdobnov, "OrthoDB v8: Update of the hierarchical catalog of orthologs and the underlying free software," *Nucleic Acids Research*, vol. 43, pp. D250–D256, Jan. 2015.

[8] L. Li, C. Stoeckert, and D. Roos, "OrthoMCL: Identification of ortholog groups for eukaryotic genomes," *Genome research*, vol. 13, no. 9, pp. 2178–2189, 2003.

[9] E. L. L. Sonnhammer and G. Östlund, "InParanoid 8: Orthology analysis between 273 proteomes, mostly eukaryotic," *Nucleic Acids Res.*, vol. 43, pp. D234–239, Jan. 2015.

[10] A. M. Altenhoff, N. Škunca, N. Glover, C.-M. Train, A. Sueki, I. Piližota, K. Gori, B. Tomiczek, S. Müller, H. Redestig, G. H. Gonnet, and C. Dessimoz, "The OMA orthology database in 2015: Function predictions, better plant support, synteny view and other improvements," *Nucleic Acids Research*, vol. 43, pp. D240–D249, Jan. 2015.

[11] D. M. Emms and S. Kelly, "OrthoFinder: Solving fundamental biases in whole genome comparisons dramatically improves orthogroup inference accuracy," *Genome Biology*, vol. 16, p. 157, Aug. 2015.

[12] R. Waterhouse, E. Zdobnov, F. Tegenfeldt, J. Li, and E. Kriventseva, "OrthoDB: The hierarchical catalog of eukaryotic orthologs in 2011," *Nucleic acids research*, vol. 39, no. suppl 1, p. D283, 2011.

[13] R. M. Waterhouse, F. Tegenfeldt, J. Li, E. M. Zdobnov, and E. V. Kriventseva, "OrthoDB: A hierarchical catalog of animal, fungal and bacterial orthologs," *Nucleic Acids Res.*, vol. 41, pp. D358–365, Jan. 2013.

[14] M. C. Munoz-Torres, J. T. Reese, C. P. Childers, A. K. Bennett, J. P. Sundaram, K. L. Childs, J. M. Anzola, N. Milshina, and C. G. Elsik, "Hymenoptera Genome Database: Integrated community resources for insect species of the order Hymenoptera," *Nucleic Acids Research*, vol. 39, pp. D658–D662, Jan. 2011.

[15] G. Suen, C. Teiling, L. Li, C. Holt, E. Abouheif, E. Bornberg-Bauer, P. Bouffard, E. J. Caldera, E. Cash, A. Cavanaugh, O. Denas, E. Elhaik, M.-J. Favé, J. Gadau, J. D. Gibson, D. Graur, K. J. Grubbs, D. E. Hagen, T. T. Harkins, M. Helmkampf, H. Hu, B. R. Johnson, J. Kim, S. E. Marsh, J. A. Moeller, M. C. Muñoz-Torres, M. C. Murphy, M. C. Naughton, S. Nigam, R. Overson, R. Rajakumar, J. T. Reese, J. J. Scott, C. R.

Smith, S. Tao, N. D. Tsutsui, L. Viljakainen, L. Wissler, M. D. Yandell, F. Zimmer, J. Taylor, S. C. Slater, S. W. Clifton, W. C. Warren, C. G. Elsik, C. D. Smith, G. M. Weinstock, N. M. Gerardo, and C. R. Currie, "The genome gequence of the leaf-cutter ant Atta cephalotes reveals insights into Its obligate symbiotic lifestyle," *PLoS Genet*, vol. 7, p. e1002007, Feb. 2011.

[16] T. H. Struck, A. R. Wey-Fabrizius, A. Golombek, L. Hering, A. Weigert, C. Bleidorn, S. Klebow, N. Iakovenko, B. Hausdorf, M. Petersen, P. Kuck, H. Herlyn, and T. Hankeln, "Platyzoan paraphyly based on phylogenomic data supports a noncoelomate ancestry of Spiralia," *Molecular Biology and Evolution*, vol. 31, pp. 1833–1849, July 2014.

[17] C. Mayer, M. Sann, A. Donath, M. Meixner, L. Podsiadlowski, R. S. Peters, M. Petersen, K. Meusemann, K. Liere, J.-W. Wägele, B. Misof, C. Bleidorn, M. Ohl, and O. Niehuis, "BaitFisher: A Software Package for Multispecies Target DNA Enrichment Probe Design," *Molecular Biology and Evolution*, vol. 33, pp. 1875–1886, July 2016.

[18] S. Nygaard, G. Zhang, M. Schiøtt, C. Li, Y. Wurm, H. Hu, J. Zhou, L. Ji, F. Qiu, M. Rasmussen, H. Pan, F. Hauser, A. Krogh, C. J. P. Grimmelikhuijzen, J. Wang, and J. J. Boomsma, "The genome of the leaf-cutting ant Acromyrmex echinatior suggests key adaptations to advanced social life and fungus farming," *Genome Res.*, vol. 21, pp. 1339–1348, Aug. 2011.

[19] Honeybee Genome Sequencing Consortium, "Insights into social insects from the genome of the honeybee Apis mellifera," *Nature*, vol. 443, pp. 931–949, Oct. 2006.

[20] R. Bonasio, G. Zhang, C. Ye, N. S. Mutti, X. Fang, N. Qin, G. Donahue, P. Yang, Q. Li, C. Li, P. Zhang, Z. Huang, S. L. Berger, D. Reinberg, J. Wang, and J. Liebig, "Genomic comparison of the ants Camponotus floridanus and Harpegnathos saltator," *Science*, vol. 329, pp. 1068–1071, Aug. 2010.

[21] C. D. Smith, A. Zimin, C. Holt, E. Abouheif, R. Benton, E. Cash, V. Croset, C. R. Currie, E. Elhaik, C. G. Elsik, M.-J. Fave, V. Fernandes, J. Gadau, J. D. Gibson, D. Graur, K. J. Grubbs, D. E. Hagen, M. Helmkampf, J.-A. Holley, H. Hu, A. S. I. Viniegra, B. R. Johnson, R. M. Johnson, A. Khila, J. W. Kim, J. Laird, K. A. Mathis, J. A. Moeller, M. C. Muñoz-Torres, M. C. Murphy, R. Nakamura, S. Nigam, R. P. Overson, J. E. Placek, R. Rajakumar, J. T. Reese, H. M. Robertson, C. R. Smith, A. V. Suarez, G. Suen, E. L. Suhr, S. Tao, C. W. Torres, E. van Wilgenburg, L. Viljakainen, K. K. O.

Walden, A. L. Wild, M. Yandell, J. A. Yorke, and N. D. Tsutsui, “Draft genome of the globally widespread and invasive Argentine ant (Linepithema humile),” *PNAS*, vol. 108, pp. 5673–5678, May 2011.

[22] J. H. Werren, S. Richards, C. A. Desjardins, O. Niehuis, J. Gadau, J. K. Colbourne, The Nasonia Genome Working Group, L. W. Beukeboom, C. Desplan, C. G. Elsik, C. J. P. Grimmelikhuijzen, P. Kitts, J. A. Lynch, T. Murphy, D. C. S. G. Oliveira, C. D. Smith, L. v. d. Zande, K. C. Worley, E. M. Zdobnov, M. Aerts, S. Albert, V. H. Anaya, J. M. Anzola, A. R. Barchuk, S. K. Behura, A. N. Bera, M. R. Berenbaum, R. C. Bertossa, M. M. G. Bitondi, S. R. Bordenstein, P. Bork, E. Bornberg-Bauer, M. Brunain, G. Cazzamali, L. Chaboub, J. Chacko, D. Chavez, C. P. Childers, J.-H. Choi, M. E. Clark, C. Claudianos, R. A. Clinton, A. G. Cree, A. S. Cristino, P. M. Dang, A. C. Darby, D. C. de Graaf, B. Devreese, H. H. Dinh, R. Edwards, N. Elango, E. Elhaik, O. Ermolaeva, J. D. Evans, S. Foret, G. R. Fowler, D. Gerlach, J. D. Gibson, D. G. Gilbert, D. Graur, S. Grunder, D. E. Hagen, Y. Han, F. Hauser, D. Hultmark, H. C. Hunter, G. D. D. Hurst, S. N. Jhangian, H. Jiang, R. M. Johnson, A. K. Jones, T. Junier, T. Kadowaki, A. Kamping, Y. Kapustin, B. Kechavarzi, J. Kim, J. Kim, B. Kiryutin, T. Koevoets, C. L. Kovar, E. V. Kriventseva, R. Kucharski, H. Lee, S. L. Lee, K. Lees, L. R. Lewis, D. W. Loehlin, J. M. Logsdon, J. A. Lopez, R. J. Lozado, D. Maglott, R. Maleszka, A. Mayampurath, D. J. Mazur, M. A. McClure, A. D. Moore, M. B. Morgan, J. Muller, M. C. Munoz-Torres, D. M. Muzny, L. V. Nazareth, S. Neupert, N. B. Nguyen, F. M. F. Nunes, J. G. Oakeshott, G. O. Okwuonu, B. A. Pannebakker, V. R. Pejaver, Z. Peng, S. C. Pratt, R. Predel, L.-L. Pu, H. Ranson, R. Raychoudhury, A. Rechtsteiner, J. G. Reid, M. Riddle, J. Romero-Severson, M. Rosenberg, T. B. Sackton, D. B. Sattelle, H. Schluns, T. Schmitt, M. Schneider, A. Schuler, A. M. Schurko, D. M. Shuker, Z. L. P. Simoes, S. Sinha, Z. Smith, A. Souvorov, A. Springauf, E. Stafflinger, D. E. Stage, M. Stanke, Y. Tanaka, A. Telschow, C. Trent, S. Vattathil, L. Viljakainen, K. W. Wanner, R. M. Waterhouse, J. B. Whitfield, T. E. Wilkes, M. Williamson, J. H. Willis, F. Wolschin, S. Wyder, T. Yamada, S. V. Yi, C. N. Zecher, L. Zhang, and R. A. Gibbs, “Functional and evolutionary insights from the genomes of three parasitoid Nasonia species,” *Science*, vol. 327, pp. 343–348, Jan. 2010.

[23] S. Richards, R. A. Gibbs, G. M. Weinstock, S. J. Brown, R. Denell, R. W. Beeman, R. Gibbs, G. Bucher, M. Friedrich, C. J. P. Grimmelikhuijzen, M. Klingler, M. Lorenzen, S. Roth, R. Schröder, D. Tautz, E. M. Zdobnov, D. Muzny, T. Attaway, S. Bell, C. J.

Buhay, M. N. Chandrabose, D. Chavez, K. P. Clerk-Blankenburg, A. Cree, M. Dao, C. Davis, J. Chacko, H. Dinh, S. Dugan-Rocha, G. Fowler, T. T. Garner, J. Garnes, A. Gnirke, A. Hawes, J. Hernandez, S. Hines, M. Holder, J. Hume, S. N. Jhangiani, V. Joshi, Z. M. Khan, L. Jackson, C. Kovar, A. Kowis, S. Lee, L. R. Lewis, J. Margolis, M. Morgan, L. V. Nazareth, N. Nguyen, G. Okwuonu, D. Parker, S.-J. Ruiz, J. Santibanez, J. Savard, S. E. Scherer, B. Schneider, E. Sodergren, S. Vattahil, D. Villasana, C. S. White, R. Wright, Y. Park, J. Lord, B. Oppert, S. Brown, L. Wang, G. Weinstock, Y. Liu, K. Worley, C. G. Elsik, J. T. Reese, E. Elhaik, G. Landan, D. Graur, P. Arensburger, P. Atkinson, J. Beidler, J. P. Demuth, D. W. Drury, Y.-Z. Du, H. Fujiwara, V. Maselli, M. Osanai, H. M. Robertson, Z. Tu, J.-j. Wang, S. Wang, H. Song, L. Zhang, D. Werner, M. Stanke, B. Morgenstern, V. Solovyev, P. Kosarev, G. Brown, H.-C. Chen, O. Ermolaeva, W. Hlavina, Y. Kapustin, B. Kiryutin, P. Kitts, D. Maglott, K. Pruitt, V. Sapojnikov, A. Souvorov, A. J. Mackey, R. M. Waterhouse, S. Wyder, E. V. Kriventseva, T. Kadowaki, P. Bork, M. Aranda, R. Bao, A. Beermann, N. Berns, R. Bolognesi, F. Bonneton, D. Bopp, T. Butts, A. Chaumot, R. E. Denell, D. E. K. Ferrier, C. M. Gordon, M. Jindra, Q. Lan, H. M. G. Lattorff, V. Laudet, C. von Levetsow, Z. Liu, R. Lutz, J. A. Lynch, R. N. da Fonseca, N. Posnien, R. Reuter, J. B. Schinko, C. Schmitt, M. Schoppmeier, T. D. Shippy, F. Simonnet, H. Marques-Souza, Y. Tomoyasu, J. Trauner, M. V. der Zee, M. Vervoort, N. Wittkopp, E. A. Wimmer, X. Yang, A. K. Jones, D. B. Sattelle, P. R. Ebert, D. Nelson, J. G. Scott, S. Muthukrishnan, K. J. Kramer, Y. Arakane, Q. Zhu, D. Hogenkamp, R. Dixit, H. Jiang, Z. Zou, J. Marshall, E. Elpidina, K. Vinokurov, C. Oppert, J. Evans, Z. Lu, P. Zhao, N. Sumathipala, B. Altincicek, A. Vilcinskas, M. Williams, D. Hultmark, C. Hetru, F. Hauser, G. Cazzamali, M. Williamson, B. Li, Y. Tanaka, R. Predel, S. Neupert, J. Schachtner, P. Verleyen, F. Raible, K. K. O. Walden, S. Angeli, S. Forêt, S. Schuetz, R. Maleszka, S. C. Miller, and D. Grossmann, "The genome of the model beetle and pest Tribolium castaneum," *Nature*, vol. 452, pp. 949–955, Apr. 2008.

[24] J. D. Thompson, D. G. Higgins, and T. J. Gibson, "CLUSTAL W: Improving the sensitivity of progressive multiple sequence alignment through sequence weighting, position-specific gap penalties and weight matrix choice.," *Nucleic Acids Res*, vol. 22, pp. 4673–4680, Nov. 1994.

[25] R. C. Edgar, "MUSCLE: A multiple sequence alignment method with reduced time and space complexity," *BMC Bioinformatics*, vol. 5, p. 113, Aug. 2004.
